# Patency of Distal Aortic Occlusion Treated With DCBs in a 13-Year-Old Child With TA

**DOI:** 10.1016/j.jaccas.2026.108374

**Published:** 2026-05-22

**Authors:** Suko Adiarto, Ayers G.I. Kalaij, Suci Indriani, Taofan

**Affiliations:** a Department of Cardiology and Vascular Medicine, Faculty of Medicine Universitas Indonesia - National Cardiovascular Center Harapan Kita, Jakarta, Indonesia; b Faculty of Medicine, Universitas Indonesia, Depok City, Indonesia

**Keywords:** children, distal aortic occlusion, drug-coated balloon, endovascular treatment, Takayasu arteritis

## Abstract

**Background:**

Distal aortic occlusion in pediatric patients with Takayasu arteritis (TA) has been very challenging to treat, especially in choosing a definitive revascularization strategy. Stenting significantly increases patency; however, the diameter might be undersized when the patient reaches adulthood.

**Case Summary:**

We reported a case of a 13-year-old girl with distal aortic occlusion caused by TA, which was successfully treated with drug-coated balloons (DCBs) in which the patency can be maintained over 2 years.

**Discussion:**

DCBs, mainly paclitaxel, help mitigate the risk of restenosis by inhibiting the proliferation of smooth muscle and intimal hyperplasia, which are both key factors of restenosis in inflammatory vascular conditions such as TA.

**Take-Home Message:**

DCBs might be a promising alternative to treat pediatric patients with TA presenting with distal aortic occlusion as it could help maintain patency while still giving chances for the arteries to grow until the patient reaches adulthood.

## Visual Summary

**Figure:**
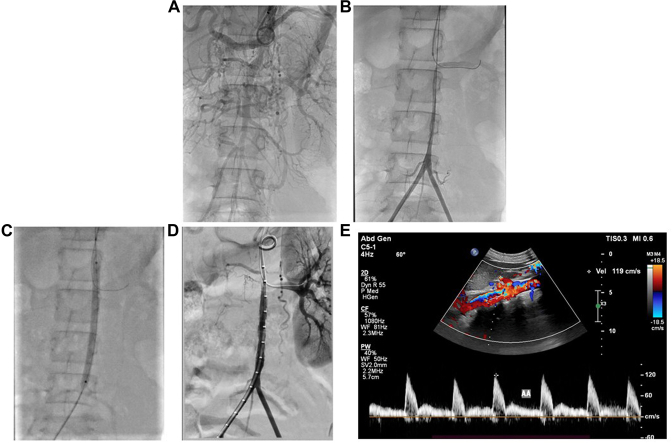

**Visual Summary:**
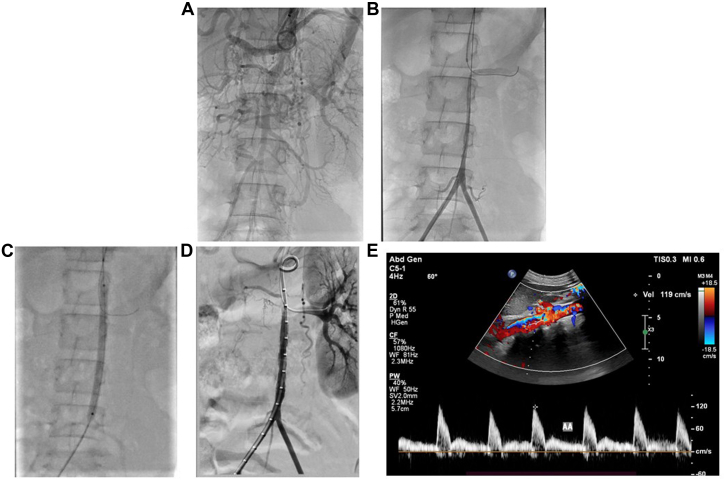
Drug-Coated Balloon Angioplasty for Distal Aortic Occlusion in Pediatric Takayasu Arteritis Maintained Patency Over 2-Years: Procedural and Follow-Up Imaging

## History of Presentation

A 13-year-old girl was referred to our hospital with the chief complaint of recurrent chest pain, headache, and claudication in both lower extremities. Her average systolic blood pressure before treatment was about 160 mm Hg. The patient had no additional symptoms.

**Take-Home Messages**

• Drug-coated balloons might be a promising alternative to treat pediatric patients with Takayasu arteritis presenting with distal aortic occlusion
• Drug-coated balloons could help maintain patency in pediatric patients with Takayasu arteritis presenting with distal aortic occlusion while still giving chances for the arteries to grow until the patient reaches adulthood.

When presented to our hospital, the blood pressure was converted to normal with a combination of antihypertensive drugs consisting of a beta-blocker, calcium antagonist, and angiotensin-converting enzyme inhibitor. Physical examination was unremarkable except for weak pulsation in both femoral arteries and a low ankle brachial index of 0.67. Four-limb blood pressure measurement was done which revealed that although the blood pressure of both lower limbs is lower (ankle brachial index: 0.67), no difference was observed between the right and left brachial pressure. Similarly, laboratory results were unremarkable except for elevated C-reactive protein (37 mg/L).

## Past Medical History

The patient had been in the care of a pediatrician in another hospital, where stage II hypertension and suspected renal artery stenosis were diagnosed. Her average systolic blood pressure before treatment was about 160 mm Hg. She underwent an abdominal duplex ultrasound in another hospital, and renal artery stenosis was suspected, resulting in secondary hypertension. The patient was then referred to our hospital.

## Differential Diagnosis

We suspected a possible diagnosis of renal artery stenosis resulting in secondary hypertension based on clinical presentations, combined with laboratory and duplex ultrasound results, which could have occurred because of inflammatory vascular conditions, one of which is Takayasu arteritis (TA).

## Investigations

**Figure 1:**
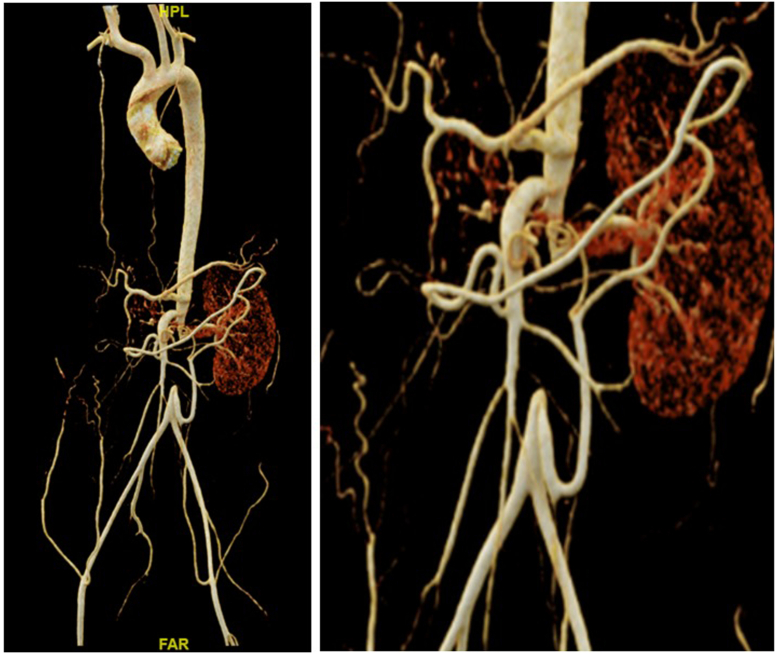
Volume Rendering Technique Computed Tomography Angiography Pre Percutaneous Transluminal Angioplasty in Distal Aortic Occlusion

Aortoiliac computed tomography (CT) angiography was then performed, which revealed a totally occluded distal abdominal aorta just below the normal left renal artery (Figure 1). The right renal artery was severely stenotic. The distal abdominal aorta was supplied by collaterals from the superior mesenteric arteries. Moreover, carotid and subclavian arteries were also screened with duplex ultrasound, whereas the pulmonary arteries were screened with CT. A coronary angiogram was also performed just after percutaneous transluminal angioplasty. All of these examinations returned with normal results.

## Management

**Figure 2:**
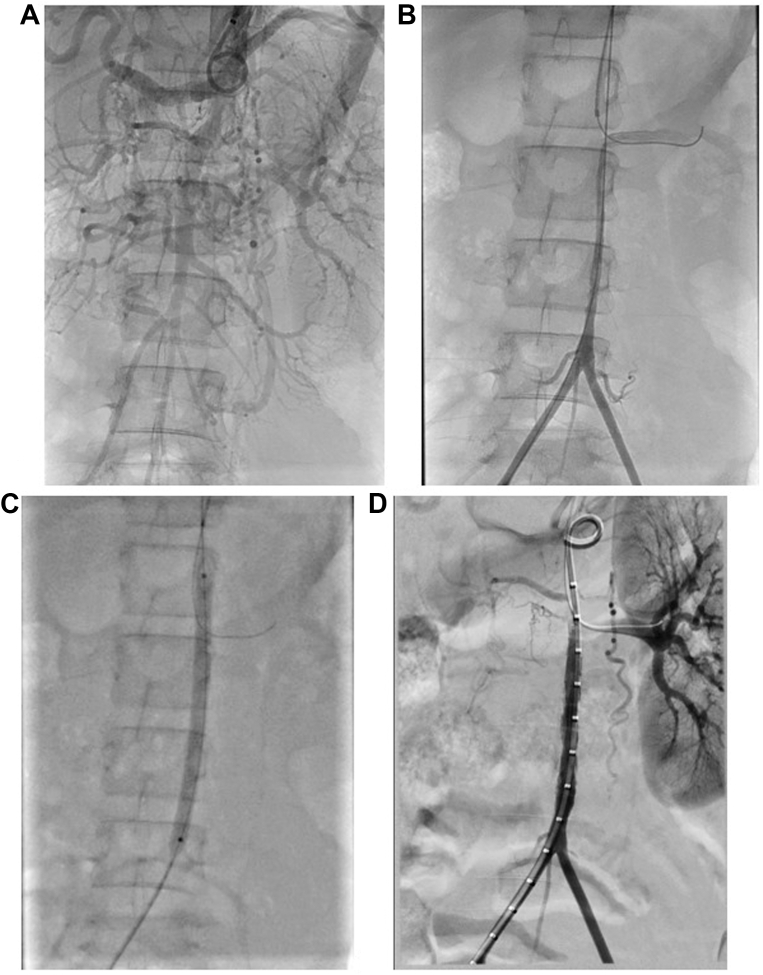
Percutaneous Transluminal Angioplasty Procedure for the Distal Aortic Occlusion (A) Aortography preprocedure; (B) Wire crossing; (C) Balloon dilatation; (D) Post ballooning.

After a multidisciplinary conference and intense discussions with the parents of the patient, we decided to perform percutaneous transluminal angioplasty. The procedure was carried out from dual accesses, including the left brachial artery and right common femoral artery. Aortogram was performed at the abdominal aorta, confirming the anatomy described by CT angiography. There was a 36-mm Hg systolic pressure gradient between the proximal abdominal aorta and the right femoral artery (Figure 2A). Both aortic CT and initial aortogram clearly showed a totally occluded abdominal aorta with extensive collateral; thus, the lesion was approached through both antegrade and retrograde approaches. The occlusion was crossed by an exchange wire followed by predilatations with a 4.0 × 80-mm balloon at 6 atm for 2 minutes and with a 6.0 × 80-mm balloon at 6 atm for 2 minutes (Figure 2B), which surprisingly crossed the occlusion retrogradely at the first attempt. Finally, dilations with 8.0 × 80-mm paclitaxel drug-eluting balloon at 4 atm were performed (Figure 2C). The final aortogram showed good results with no residual stenosis or dissection (Figure 2D). No significant systolic pressure gradient was observed. Hospitalization was uneventful, and the patient was discharged on the third postoperative day. Full details of the procedures were given through Videos 1 to 5.

## Outcome and Follow-Up

**Figure 3:**
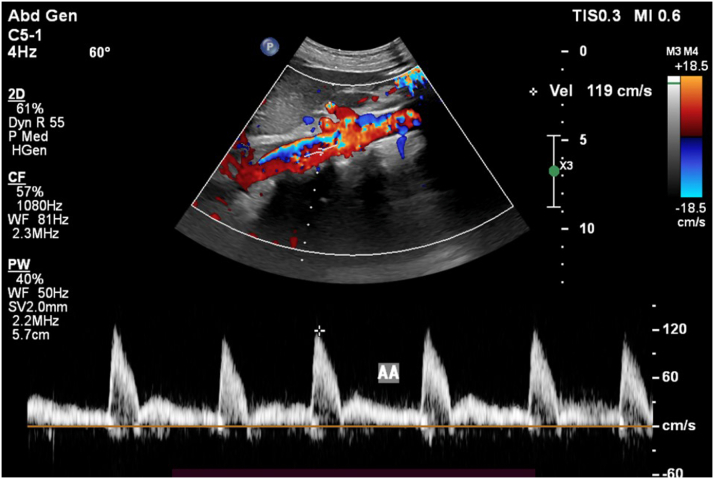
1-Year Follow-Up Post Percutaneous Transluminal Angioplasty Distal Aortic Intervention Through Duplex Abdomen Ultrasound

**Figure 4:**
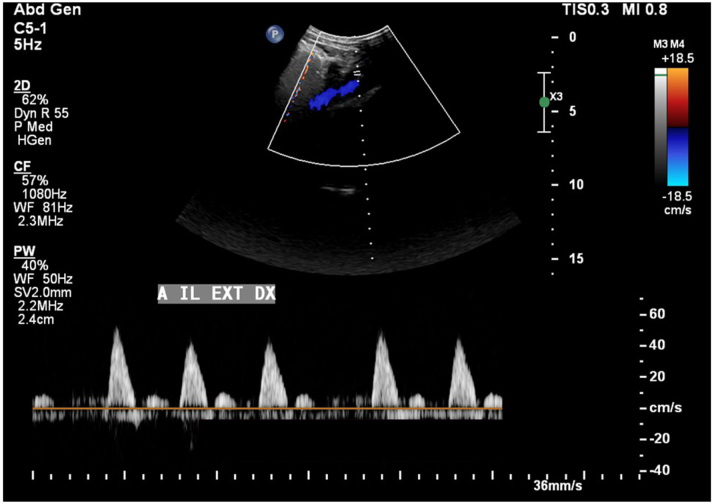
2-Year Follow-Up Post Percutaneous Transluminal Angioplasty Distal Aortic Intervention Through Duplex Bilateral Common Iliac Artery Ultrasound

Upon follow-ups, the blood pressure was normal even after discontinuation of triple antihypertensive therapies. Headache and claudication had completely disappeared. Those complaints never recurred after 1 year of follow-up. At 1-year follow-up, pulsations of both femoral arteries remained adequate. Additionally, duplex abdomen ultrasonography showed triphasic waves suggesting normal arterial flow (Figure 3). At 2-year follow-up, the patient then underwent bilateral common iliac artery ultrasound, which shows a triphasic wave (Figure 4). Furthermore, regular ultrasound follow-up for 3 years showed the abdominal aorta was still patent, which was then treated only with antiplatelet drugs and statins. No anti-inflammatory or immunosuppressive treatment was given because neither clinical nor laboratory markers of inflammation were present.

## Discussion

TA is a rare disease that usually affects large vessels, one of which is the distal aorta. The occlusion of distal aortic arteries, as seen in this 13-year-old patient, poses a challenge, considering the patient was still a pediatric patient, which has unique anatomical considerations.^1^, ^2^, ^3^, ^4^

Endovascular intervention has been a good alternative to surgical bypass in occluded or stenotic vessels resulting in organ ischemia. Despite its less invasive nature, the outcome is as effective as surgical bypass.^5^ Its patency, however, might not be as good. Although generally the problem could be solved by stent implantation, it might not be true in pediatric patients with TA,^5^ where balloon angioplasty alone resulted in a rate of restenosis of approximately 40%. Stent implantation may significantly improve the patency, but as the patient reaches adulthood, the stent might become relatively small, causing significant stenosis and leading to potential long-term risk of occlusion.^2^

Drug-coated balloons (DCBs), according to research, are effective in maintaining patency and reducing restenosis in atherosclerotic lesions. DCBs have been shown by reviews and meta-analysis as a method that delivers an antiproliferative drug, mainly paclitaxel, to the site of occlusion, which could help prevent intimal hyperplasia and restenosis, which is common in TA, especially in pediatric cases.^6^^,^^7^ DCBs have also been used for patients with TA who have thoracic great vessel stenosis^4^ and renal artery stenosis.^3^ However, the distal aortic occlusion (AO), especially in pediatric patients, has not been robustly reported.

TA is a chronic inflammatory disease that causes thickening of the vessel walls, resulting in occlusion or stenosis. The inflammatory process could lead to fibrous formation and thickening of the intima as part of vascular remodeling, which causes obstruction of blood flow. Medications such as paclitaxel or cytostatic drugs could inhibit this pathway, which theoretically prevents restenosis. Several immunological pathways, which include those by interleukin-6, tumor necrosis factor-alpha, and interferon-gamma, activate T cells and macrophages via the Janus kinase/signal transducers and activators of transcription and nuclear factor kappa-light-chain-enhancer of activated B cells pathways into granulomatous inflammation, leading to fibroblast proliferation and vascular remodeling.^8^^,^^9^ Paclitaxel interferes with all these processes: It stabilizes microtubules, which disrupts fibroblast and smooth muscle cell proliferation, and inhibits cytokine-driven pathways such as nuclear factor kappa-light-chain-enhancer of activated B cells and interleukin-6 signaling. The actions exert an influence on reducing intimal hyperplasia and preventing further AO in TA. This is seen in the patient presented in this case, where balloon angioplasty with DCBs, mainly paclitaxel, helps mitigate the risk of restenosis by inhibiting the proliferation of smooth muscles and intimal hyperplasia, which are both key factors of restenosis in inflammatory vascular conditions, and could maintain patency over 1.5 years. Paclitaxel was delivered through a balloon directly to the lesion site, therefore reducing the formation of excessive tissue, and thus, a better vessel patency in the long term could be achieved.^9^^,^^10^

Therefore, this case demonstrates that DCBs could be used for revascularization of the artery stenosis due to TA with a good patency while still giving chances for the arteries to grow according to age until reaching adulthood.

## Conclusions

This case has highlighted that DCBs might be a preferable option to treat pediatric patients with TA presenting with distal AO as it could help maintain patency while still giving chances for the arteries to grow accordingly.

## Funding Support and Author Disclosures

The authors have reported that they have no relationships relevant to the contents of this paper to disclose.

## Appendix

Video 1 Aortogram Showing Totally Occluded Abdominal Aorta With Extensive Collaterals

Video 2 Retrograde Wire Crossing of the Totally Occluded Abdominal Aorta Using Wire Crossing was surprisingly easy.

Video 3 Retrograde Aortogram Showing the Wire in Place and Collateral Vessels Filled Retrogradely

Video 4 Balloon Angioplasty With a Drug-Coated Balloon

Video 5 Final Aortogram Showing Good Antegrade Flow of Abdominal Aorta With Diminished Collaterals

## References

[1] Aeschlimann F.A., Yeung R.S.M., Laxer R.M. (2022). An update on childhood-onset Takayasu arteritis. Front Pediatr.

[2] Xiao Y., Zhou J., Wei X. (2016). Outcomes of different treatments on Takayasu’s arteritis. J Thorac Dis.

[3] Bi Y.-H., Ren J.-Z., Yi M.-F. (2019). Drug coated balloon angioplasty for renal artery stenosis due to Takayasu arteritis: report of five cases. World J Clin Cases.

[4] Nicholas W.J., Maloney T.G. (2022). Drug-coated balloon angioplasty for thoracic great vessel stenosis due to Takayasu arteritis with 1-year follow-up. J Endovascular Ther.

[5] Kumar S. (2012). Takayasu’s arteritis: paediatric perspective. Indian J Rheumatol.

[6] Prasad R.M., Mujer M., Baloch Z.Q. (2021). Efficacy, safety, and clinical outcomes of paclitaxel-coated balloon angioplasty for de-novo femoropopliteal peripheral arterial disease: a systematic review and meta-analysis of random-ized clinical trials. Medp Cardiol Vasc Med.

[7] Tataru D.-A., Lazar F.-L., Onea H.-L. (2024). Benefits and challenges of drug-coated balloons in peripheral artery disease: from molecular mechanisms to clinical practice. Int J Mol Sci.

[8] Bhandari S., Butt S.R.R., Ishfaq A. (2023). Pathophysiology, diagnosis, and management of Takayasu arteritis: a review of current advances. Cureus.

[9] Shanmugasundaram M., Murugapandian S., Truong H.T. (2019). Drug-coated balloon in peripheral artery disease. Cardiovasc Revasc Med.

[10] Gurgoglione F.L., De Gregorio M., Benatti G. (2024). Paclitaxel-coated versus sirolimus-coated eluting balloons for rercutaneous coronary interventions: pharmacodynamic properties, clinical evidence, and future perspectives. *Future Pharmacology*.

